# Long-term outcome of autistic spectrum disorder: a retrospective case study in a southern italian region

**DOI:** 10.1186/s13052-017-0399-z

**Published:** 2017-09-20

**Authors:** Francesca Felicia Operto, Federica Martino, Annalisa Rinaldi, Angelo Cerracchio, Giovanni Salvati, Mariano Orza, Claudia Lembo, Gianvito Panzarino, Claudia Di Paolantonio, Alberto Verrotti, Giovanni Farello, Giangennaro Coppola

**Affiliations:** 1 Child and Adolescent Neuropsychiatry, Faculty of Medicine and Surgery, University of Salerno, Salerno, Italy; 2 ANFAS Rehabilitation Center, Salerno, Italy; 3 Serapide Rehabilitation Center, Naples, Italy; 4 ASL Mercato S.Severino, Salerno, Italy; 5 Tiwan Rehabilitation Center, Battipaglia, Italy; 6 Department of Pediatrics, University of L’Aquila S.Salvatore Hospital Via Lorenzo Natali, 1 Coppito, 67100 L’Aquila, Italy

**Keywords:** Autism spectrum disorder, Long-term outcome, Quality of life, Social and adaptive abilities

## Abstract

**Background:**

Autism Spectrum Disorder (ASD) is a complex neurodevelopmental disorder, characterized by impaired social communication and restricted and repetitive behaviours, as well as associated features including intellectual disability and impaired sensorimotor function.

Despite a growing interest in this devastating disorder for families and young parents, there are no certainties as regards its aetiology, although a significant genetic background is considered to be important.

Since there is little information about the social adaptation and quality of life of patients with Autism Spectrum Disorder, we decided to study and evaluate the long-term outcome and quality of life in a sample of children, adolescent and young adults.

**Methods:**

This is a case study of subjects diagnosed with ASD and followed by clinics and rehabilitation centers in Campania region, in the south of Italy.

The study sample was composed by 110 patients (83 males, 27 females), aged between 8.1 and 28.0 years (mean 20.6; median 21.2; SD ± 4.85), recruited in 8 rehabilitation centers of Campania region.

A follow-up interview was performed by means of a questionnaire administered to the parents/caregivers of patients at a mean age of their son/daughter of 20.6 years (median 21.2 years; range 8.1-28.0).

**Results:**

Reports from parents or caregivers show an overall improvement with regard to social and adaptive abilities in a group of teen-agers and young adults with ASD.

Major concerns on significant quality of life parameters such as independent living, work experiences, friendships and relationships, accommodation type, recreational activities and personal autonomy were persisting.

**Conclusions:**

The present study shows an overall improvement with regard to social and adaptive abilities in a large number of subjects. Considerable problems are related to autonomy, employment opportunities and social relationships of these patients. Parents need more recreational activities and continuous support with facilities for families.

## Background

Autism Spectrum Disorder (ASD) is a complex neurodevelopmental disorder, characterized by core symptoms of impaired social communication and restricted and repetitive behaviours, as well as associated features including intellectual disability and impaired sensorimotor function [1].

Prevalence of ASD is now evaluated equal to 14.7 per 1000 (one in 68) in children aged 8 years with a male/female ratio of 4:1, with a significant increase in the last decades [2].

Despite a growing interest in this devastating disorder for families and young parents, there are no certainties as regards its aetiology, although a strong genetic background is considered to be important [3–5]. Hence, the feelings of dejection and despair of families and society (teachers, educators, speech therapists, etc.) are strengthened by the lack of a unique effective therapy.

Since there is little information about the social adjustment and quality of life in children, adolescents and young adults with ASD, we decided to study and evaluate the long-term outcome and quality of life in a sample of patients with ASD.

## Methods

This is a case study of subjects diagnosed with ASD, closely followed in clinics and rehabilitation centers in Campania region, in the south of Italy. Patients were selected according to the following inclusion criteria: 1) age (8 years and older); 2) diagnosis of ASD according to the criteria of DSM5 (more in detail, diagnoses were performed in a time-frame ranging from 1995 to 2010 by means of DSM-IV-TR criteria, and were all updated to the DSM5). Exclusion criteria were: 1) evidence of neuro-metabolic diseases and/or neurodegenerative disorders; 2) abnormal values of auditory brainstem evoked responses; 3) poor compliance to fill in the questionnaire as requested by the study protocol.

Families or caregivers of the institutionalized patients were traced through computer databases and medical records of each center. Prior to the start of the study, parents or caregivers signed an informed consent form.

Two hundred and forty-six patients who met the inclusion criteria were initially enrolled into the study; 136 of them were excluded for poor compliance in completing the follow-up questionnaire or because lost to follow-up.

The study sample was composed by 110 patients (83 males, 27 females), aged between 8.1 and 28.0 years (mean 20.6; median 21.2; SD ± 4.85) at follow-up, recruited in 8 rehabilitation centers of Campania region, in the south of Italy.

The first consultation occurred between 3 months and 7 years (mean ± SD: 2.9 ± 2.1 years).

The follow-up interview included age at follow-up, symptoms most compromising the patient’s quality of life, communication skills, school attendance and academic progression, money awareness, work experience, driving license, personal autonomy, independent living, marital status, recreational activities and friendships, crimes and law violations, rehabilitation treatments, drug-therapy, systemic diseases and surgical interventions, parents’ expectations and concerns.

The follow-up questionnaire was administered to parents/caregivers through direct interview.

## Results

Table 1 summarizes the main data that emerged from the questionnaire administered to the parents/caregivers of patients with ASD when they had a mean age of 20.6 years (median 21.2; SD ± 4.85; range 8.1-28.0).

**Table 1:** Follow-up at a mean age of 20.6 years (range 8.1-28.0)

	Relative frequency (%)
The most outcome-related symptoms according to the parents:	
Delay/absence of speech	72.2
Stereotypies	48.9
Behavioral disorder (i.e. aggressivity)	41.1
Aloneness	30
Sleep disorder	20
Eating disorder	13.3
Attention deficit	43.3
Personal autonomies	
to dress him/herself independently	40
to dress him/herself with verbal support	42.7
incapable to dress him/herself	17.3
to eat independently	79.1
to eat with verbal support	15.5
incapable to eat independently	5.4
To go out for a walk alone	13.1
Money awareness	24.8
Time spent away from home (hours/day)	
≤ 1	1,1
≥ 2 h and ≤4	16.3
≥ 5	82.6
Work experiences	6.3
Driving licence	2.1
Infrangements and social rules violations	26.3
Rehabilitation treatments	84
Psychoeducation therapy	31.5
Delacato method	24.7
Psychomotor treatment	18
Language therapy	15.7
Occupational therapy	14.6
Applied Behavioral Analysis	5.6
Water multi-systemic therapy	3.4
Psychoterapy	2.3

The overall clinical outcome after the diagnosis was referred as improved in 81.5%, unchanged in 10.9% and worsened in 7.6%.

The communication was verbal in 66%, absent in 15.1% and mimic/gestural in 18.9% (two subjects in this group communicated only through writing). Sensory perceptual skills were considered unchanged in 30.2%, improved in 58.8% and worsened in 11% of cases.

Among the most disturbing symptoms, language delay/absence (72.2%), stereotypies (48.9%), attention deficit (43.3%), behavioral disorders such as aggression (41.1%) and isolation (30%), sleep disorders (20%) and food intake problems (13.3%), were the most frequently reported.

With regard to school attendance at follow-up, 31.2% did not attend school; the others were attending classrooms in relation to their age; only 4.3% of this population reached university courses. Noteworthy, 19.2% of patients prematurely discontinued school frequency and in many cases it was sporadic.

The ability to dress was reported in 40% of cases, while eating alone was possible in 79.1% and going out for a walk alone in 13.1%. Money awareness and correct use was reported in 24.8%. The hours spent away from home were at least 5 or more a day in 82.6% of patients, while work experience (considering patients aged 14 and over) was reported in only 5 cases (6.3%).

The majority (90%) of our patients lived with their parents or relatives and 10% were institutionalized. Driving license, considering only patients aged more than 18, was achieved by one out of 48 patients (2.1%). Law violations were reported in 29 patients (26.3%), most frequently represented by running away from school, from home and aggression.

Epileptic seizures occurred in 31 patients (28.9%) with an age at onset below 4.0 years in 9 patients.

With regard to rehabilitation treatments at follow-up, no treatment was reported in 16% of patients (mean age 21.7 years); other patients (mean age 14.3 years) were under different treatments including psychomotor therapy (18%), speech/language therapy (15.7%), behavioural or psychoeducational therapy (31.5%).

When asked about the future of their son/daughter, 60.7% of the parents hypothesized a persisting need of a life with family support, while 12.4% believed in a life without support and only 4.5% in a within a protected community. Thirteen percent of parents hoped for a fully autonomous life their son. Among the parents’ major concerns, lack of social support/awareness (66.3%), poor autonomy in daily activities (62.9%), and lack of scientific knowledge on the disorder (33.7%), were more frequently reported.

## Discussion

Reports from parents or caregivers about their sons/daughters with ASD, followed in eight rehabilitation centers throughout Campania region, in the south of Italy, disclosed an overall limited independence and persisting behavioral and adaptive problems in the long term. Major concerns on significant quality of life parameters such as independent living, work experience, friendships and relationships, accommodation type, recreational activities and personal autonomy were persisting.

With respect to language skill, our data are in keeping with those reported by Kobayashi et al. [7] and Howlin et al. [8]. In our series indeed the verbal component was achieved by 66% of our patients, including a verbal ability ranging from verbal unintelligible production to a well- structured expressive language.

As for school attendance, while almost all of our patients attended public school, more than 90% of subjects with ASD from European or non European countries went to special schools [6–12], and 7-8% of subjects went to regular classes [12].

In our series, a few patients only (4.3%) reached university courses, similarly to other reports (2.5% by [7]; 7.3% by [8]; 2.1% by Eaves and Ho, 1002;; 0.8% by [12]). Noteworthy, school attendance in most of our cases was discontinuous despite the assistant teacher.

Personal autonomy, including daily activities like to eat, to dress and to wash without any help was reported much less frequently in our series than in other studies [11]. One of the reason might be a parental overprotective and anticipatory educational style in our country. Recreational activities and friendships were also rarely reported in our study, the mean amount of 5 h a day spent outside being mostly for rehabilitation activities. On the other side, Billstedt et al. [12] reported involvement in regular leisure activities such as horse riding, bowling and swimming in 33% of their patients and specific interests with an outstanding variation of topics in about 90% of them.

Five of our patients (aged 14 and over) had a short lasting work experience, in keeping with [12]. More encouraging data come from Howlin et al. [8], who reported a working activity in approximately one-third of their 68 adult patients (mean age at follow-up 29 with an IQ total score over 50). Other authors, such as Howlin et al. [8] and Kobayashi et al. [7], reported a working activity in 20.8% of patients, with an average age of 21.5 years.

In both series from Kobayashi et al. [7] and Howlin et al. [8], a higher percentage of patients with an occupational activity were related to higher intellectual and adaptive social functioning. The positive correlation between work experiences and a better intellectual functioning was also found by Cederlund et al. [10] and by Eaves and Ho [11] who reported that 56% of young adults with ASD (mean age 24) had been occupied at least once in their life in unpaid or part-time job, for an average of 5 h a week.

Finally, as underlined by Kobayashi et al. [7], the concomitant economic conditions also concur to provide good opportunities for these individuals to find a job.

**Fig. 1:**
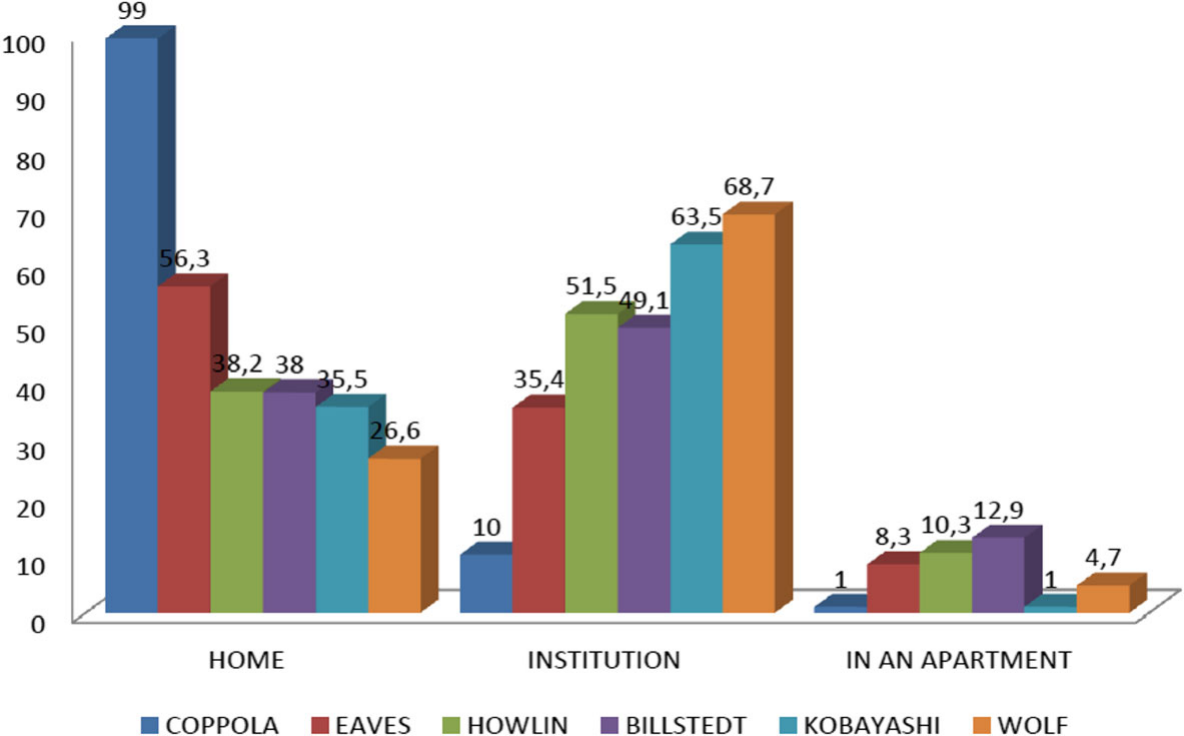
Percentage of patients who lived at home, in an institution and in an apartment in the various studies

A high percentage of our patients lived with their parents or relatives. This trend may have been influenced by the average age of our patients that was under 25 years. A tendency to keep these patients institutionalized (institutions, home groups, residential centers) more frequently recurs in other series (Fig. 1). Overall, patients able to live by themselves are very few in all the studies (0.9 to 8.3%), almost exclusively those with high-functioning Asperger syndrome, as Cederlund et al. [10] described. It should be underlined that virtually all these subjects while living on their own still needed an external support from their parents.

Antisocial behaviors, infringements and social rule violations were reported more frequently in our cases (26%) than in other series (i.e. 3% by [13]). The younger age of our cases and the higher prevalence of living school or home could in part contribute to explain such a difference.

Speech language was reported more frequently in our cases (26%) than other series (i.e. 3% by [13]). It was reported in the questionnaire as the most significant outcome-related factor soon after the global socio-communicative ability. Sleep and nutrition problems were also considered paramount for the quality of life and the adaptive functioning of these patients.

Only a few parents believed in a potential autonomous life of their son/daughter. Others had begun to evaluate the possibility for their son to live in a community, although with conflicting feelings. A few others chose not to answer, unable to imagine a given life condition for their beloved son after the end of their own life.

In this context, the parents showed the greatest concern about the lack of autonomy in daily activities and the lack of people’s support/knowledge of these issues. As regarding this topic, literature is encouraging, because the lack of knowledge regarding autism only concerns the 27% of the parents. Accordingly, Eaves and Ho [11] report a global satisfaction on health care and community support respectively by 80 and 65% of the parents, as well as a favourable evaluation by half of the parents for the institutional support and facilities to find a job to their child. On the other hand, Billstedt et al. [12] confirm that a major concern of parents is represented by not being able to offer a meaningful occupation or daily activities to their child, and underlines the need for life-long institutional support and education for adults with autism, as well as the need for respite care for families.

As to the overall clinical situation at follow-up with respect to the period when the diagnosis of ASD was first delivered, it was reported as improved in most of our cases, in keeping with other studies [7, 8, 11, 12], based on the ability to live independently or with a job, living at home with support or with a certain degree of autonomy. Furthermore, Kobayashi et al. [7] found a significant positive correlation between language and IQ level at 6 years of age and level of adaptive functioning at follow-up (average age 21.5 years).

### Limitations and strengths of the study

One major limitation of this study it is the retrospective diagnosis of ASD; the diagnosis was based mainly on the criteria of the DSM-IV-TR. In all cases, however, diagnosis was re-evalued following DSM5 criteria, in patients who are well known and followed long time by physicians well experienced in the field of social-communication and autistic spectrum disorders.

## Conclusions

In conclusion, the present study shows an overall improvement with regard to social and adaptive abilities in a group of adolescent and young adults with ASD. Lack of autonomy, employment opportunities and social relationships represents a persistent burden of care for their families.

Noteworthy, parents expressed a strong need for more recreational activities and continuous support with facilities for families aimed at solving practical problems and daily welfare of their sons.

## Abbreviations

ABA: Applied behavior analysis
ASD: Autistic spectrum disorder
DSM-IV-TR: Diagnostic and statistical manual of mental disorders text revision
MMWR: Morbidity and mortality weekly report
SD: Standard deviation
